# Combined Ravitch and Nuss procedure for pectus excavatum with dyspnea following scoliosis repair

**DOI:** 10.1093/jscr/rjad618

**Published:** 2023-11-11

**Authors:** Naoyuki Oka, Kyohei Masai, Yu Okubo, Kaoru Kaseda, Tomoyuki Hishida, Keisuke Asakura

**Affiliations:** Division of Thoracic Surgery, Department of Surgery, Keio University School of Medicine, Tokyo, Japan; Division of Thoracic Surgery, Department of Surgery, Keio University School of Medicine, Tokyo, Japan; Division of Thoracic Surgery, Department of Surgery, Keio University School of Medicine, Tokyo, Japan; Division of Thoracic Surgery, Department of Surgery, Keio University School of Medicine, Tokyo, Japan; Division of Thoracic Surgery, Department of Surgery, Keio University School of Medicine, Tokyo, Japan; Division of Thoracic Surgery, Department of Surgery, Keio University School of Medicine, Tokyo, Japan

**Keywords:** pectus excavatum, scoliosis, dyspnea, Nuss procedure, Ravitch procedure

## Abstract

Pectus excavatum (PE) is often associated with scoliosis and can elicit cardiovascular disturbances under rare conditions. Here we report a patient who was treated with a combined Ravitch and Nuss procedure for PE with dyspnea following scoliosis repair to improve her symptoms. The patient was a 49-year-old woman with a history of PE and scoliosis. Right inferior pulmonary vein stenosis was caused by posterior spinal fusion for scoliosis prior to the PE repair. We could safely correct the chest wall deformity and treat dyspnea by performing a modified Ravitch repair in combination with the Nuss procedure.

## Introduction

Pectus excavatum (PE) is the most common congenital chest wall deformity [1]. It sometimes elicits cardiovascular disturbances and pulmonary dysfunctions as a result of severe sternal depression [2]. Therefore, surgeons must consider the risk of serious complications during surgical treatment of severe chest deformities. In several reports, scoliosis was identified in patients with PE [3, 4], and surgical correction could be necessary for both PE and scoliosis repair. However, because a limited number of studies have reported patients who underwent surgery for PE and scoliosis, the treatment modality and surgical management for patients with both PE and scoliosis remain unclear. We report here a case of PE who developed dyspnea following surgery to correct scoliosis.

## Case report

The patient was a 49-year-old woman with a history of PE and scoliosis since she was 10 years of age. She presented with a 2-year history of dyspnea and was diagnosed with scoliosis-induced ventilatory impairment. She underwent posterior spinal fusion for the management of scoliosis. However, her dyspnea worsened 1 year postoperatively.

**Figure 1:**
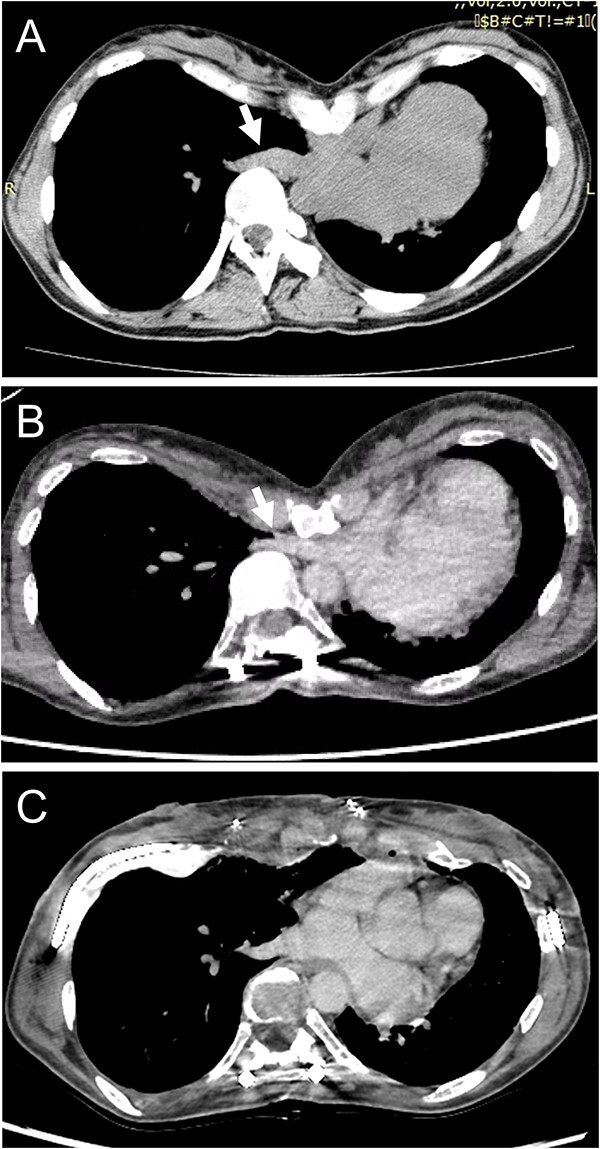
(**A**) Chest CT shows no compression of the right inferior pulmonary vein (IPV) before scoliosis repair (arrow). (**B**) Severe right IPV stenosis is induced by posterior spinal fusion (arrow). (**C**) Postoperative chest CT indicates that right IPV stenosis is released by elevation of the depressed sternum.

A spirometry test before surgery showed a vital capacity (VC) of 2.0 L (76% of predicted). After surgical repair of her scoliosis, VC was 1.7 L (66% of predicted) and she experienced ventilatory impairment. As part of the imaging test, a chest X-ray demonstrated thoracic scoliosis with a Cobb angle of 62° before surgery, and the curve improved due to a posterior correction of the scoliosis. On the other hand, chest computed tomography (CT) revealed that severe chest excavation presented a lower sternum as an extreme point, and the depressed sternum compressed cardiovascular structures. Notably, a preoperative chest CT indicated that there was no compression of the right inferior pulmonary vein (IPV) (Fig. 1A); however, after scoliosis surgery, severe right IPV stenosis occurred secondary to narrowing of the space between the 9th thoracic vertebrae, which were shifted toward the midline following posterior spinal fusion and lower sternal depression as a result of PE (Fig. 1B). An echocardiogram showed that the systolic right inferior pulmonary venous flow (PVF) increased from 42.5 cm/s to 135 cm/s during changing position.

According to these imaging results, exacerbation of dyspnea was attributed to a ventilation-perfusion mismatch because of severe right IPV stenosis. We planned to perform surgery for PE repair to achieve surgical release of the IPV stenosis. The patient underwent a Combined Ravitch and Nuss (CRN) procedure. Thoracoscopy revealed IPV distention due to compression by the severely depressed sternum. The sternal depression was corrected with separation of the 4th–7th costal cartilages, followed by sternal elevation using two pectus bars. She was discharged on the 11th postoperative day without any complications. A postoperative chest CT exhibited an improvement of sternal depression and the right IPV stenosis (Fig. 1C). An echocardiogram revealed that the systolic PVF changed from 49.7 cm/s to 75.7 cm/s during postural changes, and the increase in PVF was mitigated, so that her shortness of breath was relieved postoperatively.

## Discussion

**Figure 2:**
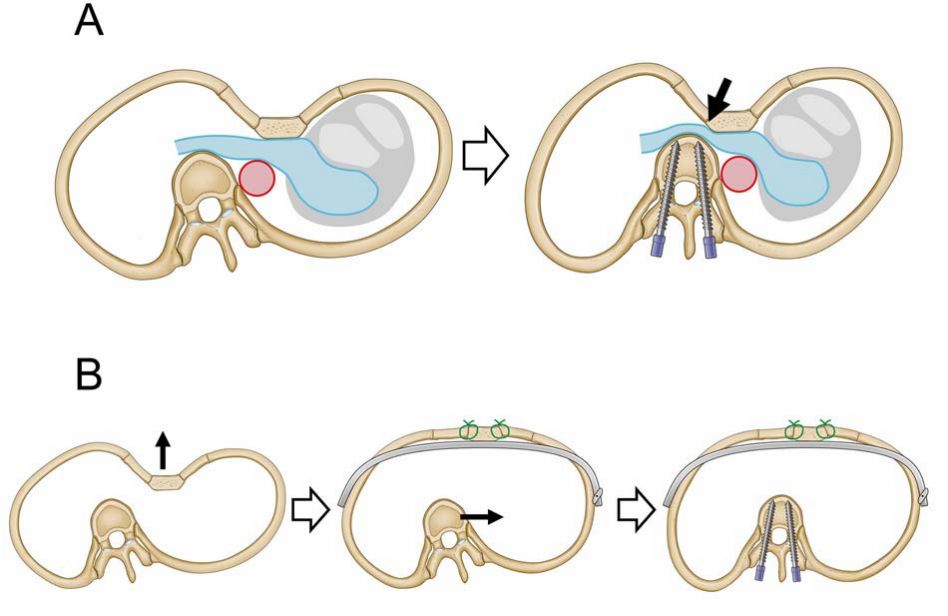
Schematic images of treatment for PE and scoliosis. (**A**) The right inferior pulmonary vein is sandwiched between the depressed sternum and the thoracic vertebrae, which moved toward the midline due to scoliosis surgery (arrow). (**B**) PE repair following scoliosis repair makes it possible to secure the distance between the sternum and thoracic vertebrae.

We performed a CRN procedure in a patient with PE with dyspnea following scoliosis repair to improve her symptoms. In previous reports, scoliosis was found in ~20% of patients with PE [2–4]. In the present case, the development of dyspnea was considered to be caused by the scoliosis repair prior to PE repair followed by worsening of right IPV stenosis (Fig. 2A). Tauchi *et al*. [5] stated that, among patients who underwent surgical treatment for scoliosis associated with PE, none experienced severe hypotension or cardiopulmonary dysfunction intraoperatively, while the distance between the depressed sternum and thoracic vertebrae was shortened postoperatively in 11 of the 20 patients. Furthermore, another report described a case of severe hypotension associated with intraoperative prone positioning in a patient with PE undergoing posterior spinal fusion for scoliosis [6]. Therefore, we should pay attention to the presence of cardiovascular disturbances in patients with severe PE and scoliosis during the perioperative period. Meanwhile, the prior authors also reported that they could safely perform posterior spinal fusion for scoliosis without perioperative complications because of surgery for PE prior to scoliosis repair [6]. Taken together, these findings suggest that if surgical repair of PE is performed first and maintains the distance between the sternum and thoracic vertebrae before scoliosis surgery [Fig. 2(B)], it is possible to avoid postoperative cardiovascular disturbances following scoliosis repair. Moreover, in a patient with sternal depression compressing cardiovascular structures, there is a risk of intraoperative cardiovascular injury because the introducer is inserted into the anterior mediastinum without the direct visualization of mediastinal organs. In such a situation, elevation of the anterior chest before passage of the introducer across the anterior mediastinum is effective for preventing cardiac perforation [7]. Additionally, the correction of thoracic deformity in adults may require more force than that in adolescents due to the greater rigidity of the adult thorax [8]. Therefore, surgical mobilization of the anterior chest wall is necessary for favorable elevation of the sternum. In our case, because the modified Ravitch repair, which improved the flexibility of the chest wall by dividing costal cartilages, was performed prior to the Nuss procedure, we could elevate the sternum safely and favorably.

We introduced the CRN procedure into PE repair only recently, and its long-term outcome is still unknown. Although the optimal timing of bar removal has not been clearly defined, since a relapse of sternal depression may elicit IPV stenosis, the bar should be kept in place for a long time to avoid the recurrence of PE.

In conclusion, we performed the CRN procedure for PE with dyspnea following scoliosis repair. PE is often associated with scoliosis, and surgical management for PE should precede the correction of scoliosis to avoid postoperative cardiovascular disturbances.

## Data Availability

All data (of the patient) generated during this study are included in this published article and its supplementary information files.
